# Nanodiamond-Enabled Passively *Q*-Switched Solid-State Laser Operating at 2 μm

**DOI:** 10.3390/nano16201313

**Published:** 2026-10-17

**Authors:** Zhe Meng, Yang Zhang, Haoyang Tang, Yuejin Jiang, Zhaoxue Li, Xining Yang, Sujian Niu

**Affiliations:** Xinjiang Key Laboratory for Luminescence Minerals and Optical Functional Materials, School of Physics and Electronic Engineering, Xinjiang Normal University, Urumqi 830054, China; meng13688626273@163.com (Z.M.); 19887079834@163.com (Y.Z.); tang_haoyang_wl@163.com (H.T.); 17881085182@163.com (Y.J.); zhaoxue_l@163.com (Z.L.)

**Keywords:** nanodiamond, saturable absorber, passively *Q*-switched, solid-state laser

## Abstract

This work demonstrates a passively *Q*-switched (PQS) Tm:YAlO_3_ (Tm:YAP) laser at 2 μm employing a nanodiamond (ND) saturable absorber (SA). Microstructural analysis indicates that the spin-coated ND film exhibits spherical grains and forms effective interfacial contact with the CaF_2_ substrate. Employing a 5%-transmittance output coupler (OC), the ND-based SA facilitated the generation of a PQS laser. Key performance metrics include: an average output power of 367.7 mW at an absorbed pump power of 9.66 W, and a central wavelength of 1931.2 nm. Moreover, stable pulse trains exhibited a minimum pulse duration of 376.9 ns at a repetition rate of 86.27 kHz, setting a record for the shortest pulse width ever reported for ND-based PQS lasers. Under the same conditions, the beam quality factors were measured as *M 2 x* = 1.02 and *M 2 y* = 1.08. These results confirm that ND serves as an excellent modulator, showing great potential for mid-infrared pulsed laser applications.

## 1. Introduction

The rapid progress of 2 µm laser technology in optical communications, biomedical imaging, and light detection and ranging has imposed increasingly stringent demands on laser performance, particularly in terms of pulse stability, peak power, and repetition rate [1,2,3,4,5]. The passively *Q*-switched (PQS) technique has emerged as a primary technique for generating high-energy pulses, benefiting from its simple configuration, low cost, and high operational stability, while aligning well with current trends toward miniaturization and system integration [6,7,8]. As the core component of PQS lasers, the saturable absorber (SA) requires no external electronic control, offers a compact structure and low fabrication cost, and directly governs the output pulse quality. Traditional SA materials, including black phosphorus (BP), transition metal dichalcogenides (TMDs), and MXenes, have been extensively adopted in PQS lasers [9,10,11,12]. However, they are still plagued by issues such as complex preparation routes, poor ambient stability, or limited operational bandwidth, which hinder their ability to satisfy high-performance laser requirements [13].

To overcome the inherent limitations of conventional SA materials, researchers have turned to carbon-based nanomaterials. Benefiting from their excellent electrical and optical properties, these materials have shown broad application prospects in photonics and optoelectronics in recent years [14]. To date, carbon-based nanomaterials have been successfully implemented in PQS fiber lasers, where they have demonstrated outstanding performance in generating ultrashort pulses ranging from sub-picosecond to nanosecond durations. Among the various carbon-based nanomaterials, graphene stands out as a representative SA for 2 μm *Q*-switched lasers owing to its zero-bandgap linear energy band structure and broadband saturable absorption covering the 1.8–2.2 μm range [15]. In practical applications, however, graphene-based SAs are constrained by oxidative degradation in air and a relatively low single-pulse damage threshold, which makes it difficult to meet the long-term reliability demands of high-average-power solid-state lasers [16]. Carbon nanotubes (CNTs) offer another alternative; they can achieve a high modulation depth at specific wavelengths, yet they suffer from poor dispersion uniformity and irreversible damage caused by photothermal accumulation [17,18,19]. Given these limitations, nanodiamonds (NDs) have emerged as a compelling alternative, offering distinct advantages as highly robust SAs. These advantages stem from their ultrahigh chemical inertness, high thermal conductivity, and good optical transparency in the visible to mid-infrared range [20]. As a representative class of carbon-based nanomaterials [21,22], NDs are not only utilized in fields such as quantum sensing and targeted drug delivery but have also been shown to effectively suppress intensity noise in optical communication links [23]. This capability is attributed to their low excitation threshold, sub-picosecond nonlinear response time, and strong third-order nonlinear coefficient [24]. Furthermore, the optical limiting behavior of NDs arises from the synergistic effects of nonlinear scattering, two-photon/stimulated Raman absorption, and nonlinear refraction [25,26]. Under moderate excitation intensities, however, detonation-synthesized NDs can exhibit saturable absorption. Detonation-synthesized NDs dispersed in aqueous solution have been demonstrated to exhibit saturable absorption under excitation by 795 nm femtosecond laser pulses, achieving a modulation depth of 12.3% and a saturation fluence of 0.18 J/cm^2^ [27]. This saturable absorption behavior originates from localized intermediate bandgap states formed by vacancies and sp^2^-hybridized carbon shells on the ND surface. These states rapidly capture and relax photogenerated charge carriers, yielding an ultrafast recovery time on the picosecond scale—thereby fulfilling the critical requirements of nanosecond *Q*-switched lasers for SA performance. ND-SA contains surface defects (sp^2^ carbon, vacancies, grain boundaries) that introduce intermediate bandgap levels for saturable absorption. Low intracavity intensity leads to carrier trapping, high cavity loss and inhibited lasing. Accumulated population inversion in Tm:YAP increases intracavity intensity and saturates the defect states; the resultant rapid loss reduction initiates avalanche oscillation and PQS pulses. NDs’ picosecond recovery is sufficiently fast for pulse generation, yet the pulse width is determined by cavity photon lifetime, Tm:YAP upper-level lifetime, initial inversion, SA parameters and output coupling, explaining the submicrosecond pulse formation and compression [28,29,30].

After clarifying the advantages of nanodiamonds (NDs), we focus on the application background of 2 μm solid-state lasers. Tm:YAlO_3_ (Tm:YAP) crystals, owing to their high thermal conductivity, broad emission bandwidth, and excellent mechanical strength, have become ideal gain media for high-power laser systems [31]. However, the development of PQS lasers based on Tm:YAP has been constrained by the lack of SAs that simultaneously possess a high damage threshold, environmental stability, and process compatibility. Although carbon nanotubes and graphene films have been tested, their performance is often compromised by thermal deformation, interfacial delamination, or oxidation failure [32,33]. Notably, although nanodiamonds have been successfully applied in erbium-doped and ytterbium-doped fiber lasers, the feasibility of fabricating ND films by spin-coating and integrating them into Tm:YAP solid-state laser cavities has not been previously reported [34].

In this study, a pulsed Tm:YAP laser was achieved by employing a spin-coated ND-based SA. The ND film’s nonlinear optical response was comprehensively evaluated. Stable pulsing operation delivered a minimum pulse width of 376.9 ns at a repetition frequency of 86.27 kHz. To the best of our knowledge, this work represents the first implementation of a PQS all-solid-state laser operating at 2 μm using NDs as the SA. These results underscore the promise of NDs as low-cost, high-damage-threshold modulators for mid-infrared pulsed laser systems.

## 2. Preparation and Characterization of the ND

### 2.1. Fabrication Process

**Figure 1:**
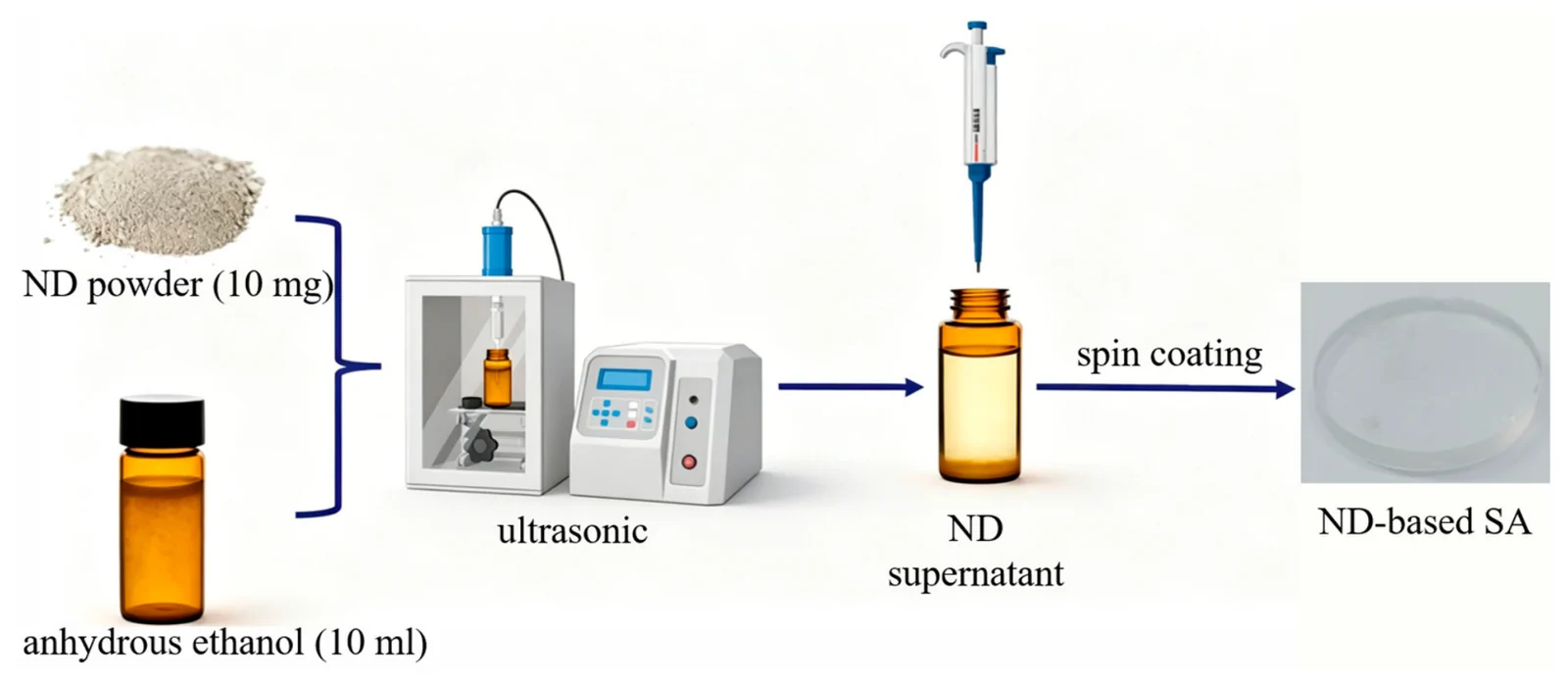
Overview of the ND-SA synthesis procedure.

ND powder (5–10 nm particle size, grayish-white, Xian Feng Nanomaterials Technology Co., Ltd., Nanjing, China) was used as the starting material. The fabrication process (Figure 1) consisted of the following steps. First, 10 mg of ND powder was dispersed in 10 mL of anhydrous ethanol (>99.7% purity) within a clean glass vial. Second, the mixture was ultrasonicated for 12 h using a cell disruptor (JY96-IIN, LICHEN, Shanghai, China) to overcome van der Waals forces and achieve uniform dispersion. Third, the suspension was left to stand at room temperature for 24 h, allowing large agglomerates to sediment; the upper homogeneous supernatant was then collected. Fourth, employing a KW-4A spin coater (KW-4A, SETCAS, Beijing, China), the supernatant was spin-coated onto a CaF_2_ (uncoated, 2.5 mm thick) substrate through three consecutive steps: 500 rpm/5 s, 1000 rpm/10 s, and 3000 rpm/15 s. The ND film was deposited only on one side of the substrate. Finally, the coated substrate was passively dried in a dust-free environment at room temperature for 12 h to fully evaporate the ethanol, yielding a uniform ND film on the CaF_2_ surface.

### 2.2. Properties Characterization

**Figure 2:**
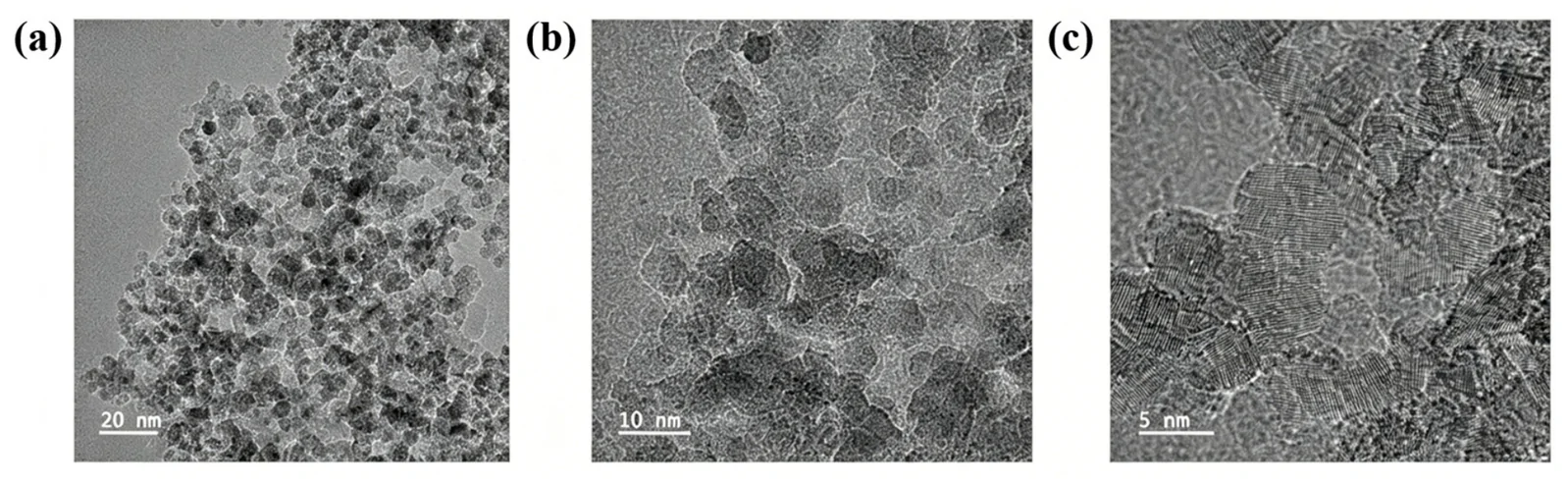
TEM images of the ND-based SA at different magnifications. (**a**) 20 nm; (**b**)10 nm; (**c**) 5 nm.

The morphology and microstructure of the ND-based SA were investigated via transmission electron microscopy (TEM, JEM-F200, JEOL, Tokyo, Japan). The low-magnification TEM image in Figure 2a reveals that the sample consists of numerous near-spherical nanoparticles with an average particle size of 4–6 nm, which aggregate in the form of soft agglomerates with clear boundaries, and no obvious sintering necks or hard agglomerates are observed. The medium-magnification TEM image in Figure 2b further confirms a narrow size distribution and well-defined contours of the particles, with no detectable thick amorphous carbon coating layer. The TEM images in Figure 2a,b further confirm that the collected ND particles have a narrow size distribution of 4–6 nm. The lattice fringes visible in the high-resolution TEM image of Figure 2c are assigned to the diamond (111) plane, featuring a spacing of approximately 0.206 nm [35]. The fringes are continuous without interruption, and no significant graphitization or structural defects are observed, indicating high crystallinity and good phase purity of the sample.

**Figure 3:**
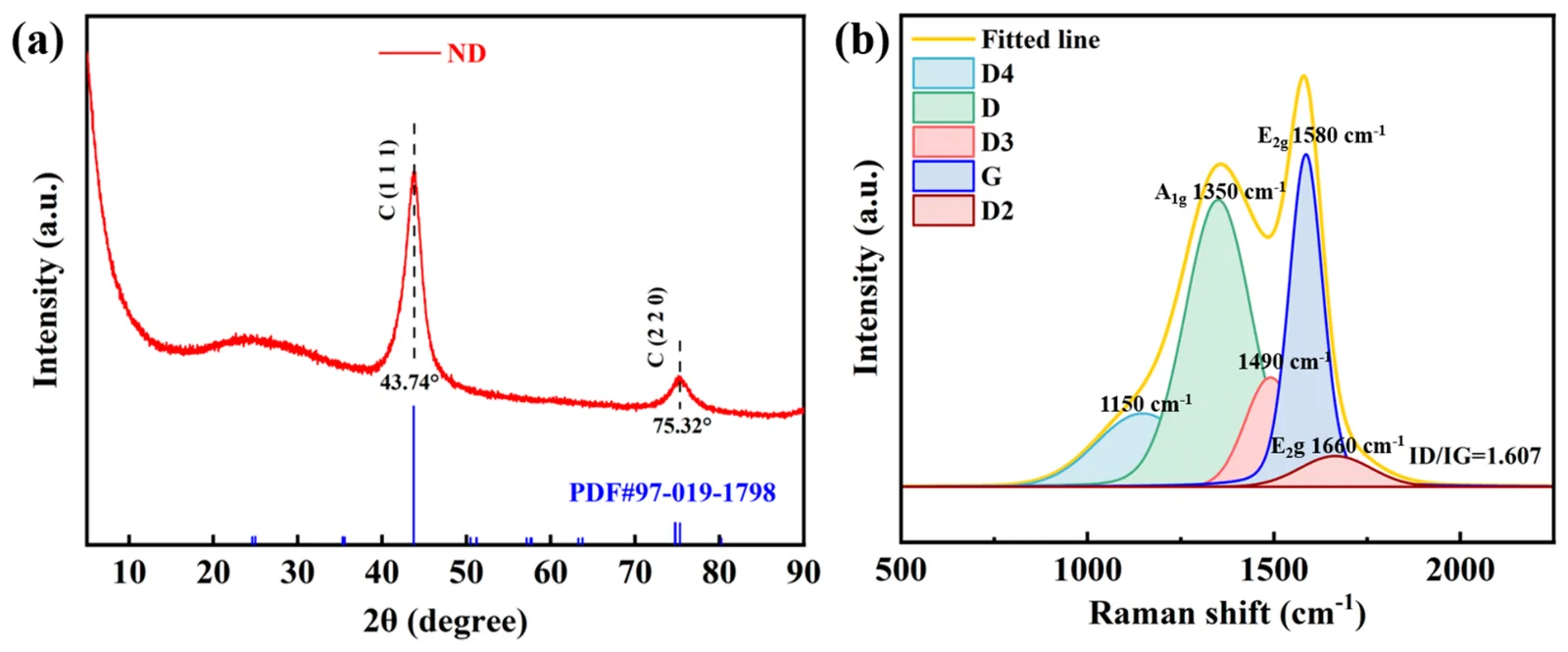
(**a**) XRD pattern of the ND powder; (**b**) Raman spectrum of the ND powder.

As displayed in Figure 3a, the crystal structure of the ND powder was characterized by X-ray diffraction (XRD, SmartLab SE, Rigaku, Akishima, Tokyo, Japan). The diffraction pattern exhibits two prominent peaks at 43.74° and 75.32°, which were assigned to the (111) and (220) planes of cubic diamond, respectively, matching well with PDF card No. 97-019-1798. The high intensity and sharpness of these characteristic peaks indicate that the NDs possess excellent crystallinity and high phase purity. Moreover, no diffraction peaks corresponding to graphite or other carbonaceous impurities were detected across the entire XRD pattern, further confirming that the sample consists predominantly of the diamond phase. Collectively, these structural analyses attest to the excellent quality of the ND, a necessary condition for realizing a high-performance optical SA [36].

Raman spectroscopy (LabRAM HR Evolution, Horiba, Kyoto, Japan) was employed to examine the structural characteristics of the ND powder (Figure 3b). The sharp diamond peak at ~1332 cm^−1^, typical of bulk diamond, is significantly broadened and weakened due to the phonon confinement effect in nanodiamonds (average size: 4–6 nm) [21]. Instead, several Raman bands associated with surface sp^2^ carbon appear. The D4 band at 1150 cm^−1^ and the D3 band at 1490 cm^−1^ are attributed to trans-polyacetylene chains at grain boundaries or on the diamond surface, with a possible minor contribution from amorphous carbon to the latter [37]. At 1350 cm^−1^, the D band arises from the A_1_g breathing mode of disordered graphite featuring edge and vacancy defects; the G band at 1580 cm^−1^ corresponds to the E_2_g stretching mode of sp^2^-hybridized carbon [38]. The D2 band at ~1660 cm^−1^ is assigned to the E_2_g mode of edge and vacancy defects in disordered graphite, which are commonly observed in disordered carbon materials [39]. The integrated intensity ratio ID/IG is 1.607, indicating a high density of defects (edges and vacancies) in the surface graphitic layer. Notably, these defects probed by Raman spectroscopy reside in the top few atomic layers of sp^2^ carbon, whereas the clear lattice fringes observed by high-resolution TEM correspond to the diamond core. The extreme thinness of the defect layer (<1 nm) prevents it from being resolved in TEM, making the two results complementary rather than contradictory. Such defect states introduce intermediate energy levels within the bandgap, acting as traps for photogenerated carriers. Upon reaching an incident intensity threshold, these defect states become saturated, switching the material from absorption to transmission and enabling the saturable absorption effect. Hence, these surface defect structures constitute the key mechanism for PQS in NDs [40].

## 3. Experimental Setup

**Figure 4:**
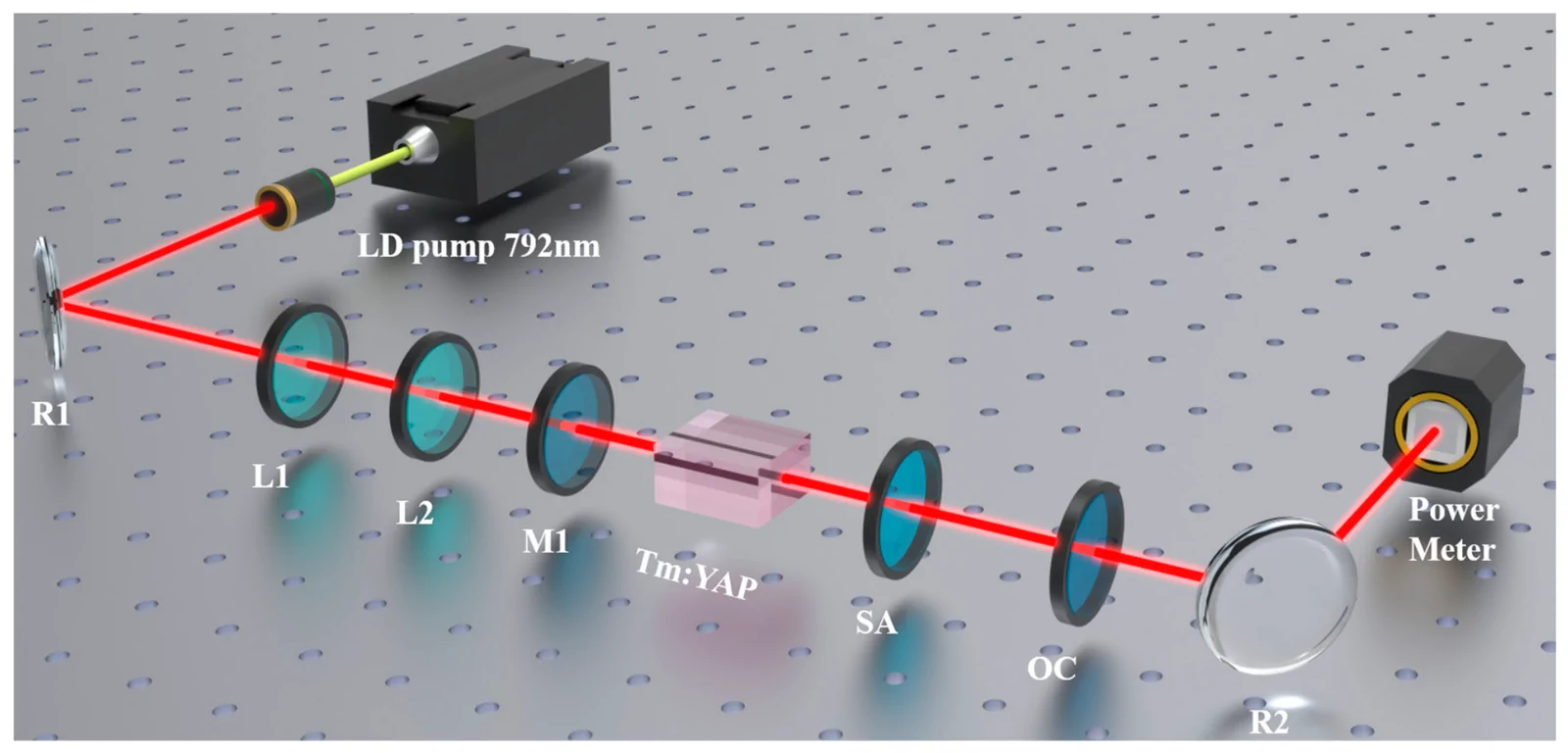
Setup for the PQS Tm:YAP laser using the ND-based SA.

Nonlinear optical characterization of the ND-based SA was performed using a PQS laser setup, as illustrated in Figure 4. At room temperature, a fiber-coupled laser diode (LD) emitted a pump power of up to 30 W at 792 nm, with the fiber characterized by a 105 μm core diameter and a numerical aperture of 0.22. The gain medium was a *b*-cut Tm:YAP crystal (3 mm × 3 mm × 7 mm, 3 at% Tm^3+^) with anti-reflection coatings on both end faces for 790–810 nm and 1900–2100 nm. The crystal, wrapped in indium foil, was mounted in a water-cooled copper heat sink for efficient thermal management. A compact short cavity was adopted for pulsed operation. Lenses L1 (*f* = 25 mm) and L2 (*f* = 30 mm) collimated and focused the pump beam, respectively. After calculating, the focused pump spot diameter was estimated to be approximately 126 μm, with a corresponding Rayleigh range of approximately 15.7 mm (Gaussian waist radius *w*_0_ ≈ 63 μm at *λ* = 792 nm). Two 45°-tilted mirrors (R1, R2) were highly reflective for both the pump and laser wavelengths. The laser cavity (total physical length: 23 mm) was composed of a plano-concave input mirror (M1) and a plane output coupler (OC). M1 had a 200 mm radius of curvature; both sides were highly transmissive over 790–810 nm, while the concave side was highly reflective from 1900 to 2100 nm. The OC was a custom-coated flat mirror, transmitting 2% or 5% at the lasing wavelength (1900–2100 nm) while remaining highly reflective at the 792 nm pump wavelength, with a typical transmittance below 0.5%. The ND thin film on CaF_2_ was inserted as the SA between the Tm:YAP crystal and the OC. Pulsing was achieved by adjusting the mirror spacing. A calibrated Thorlabs power meter (model PM100D console, DC-100 kHz, Thorlabs, Newton, NJ, USA) equipped with an S425C thermopile sensor (190 nm–20 μm spectral range) was used to monitor and record the average output power.

## 4. Results and Discussion

**Figure 5:**
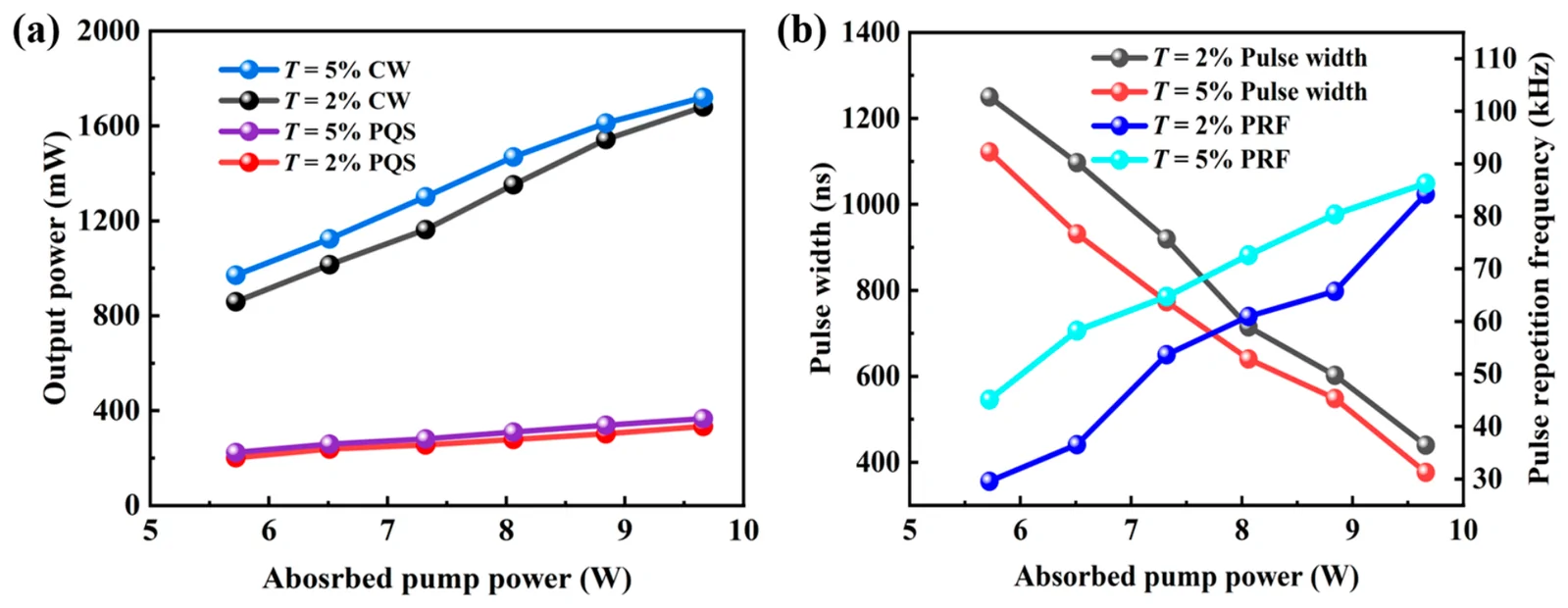
Laser performance using an ND-based SA. (**a**) Output power vs. absorbed pump power at OC transmissions of 5% and 2% for CW and PQS modes; (**b**) pulse width and repetition rate versus absorbed pump power.

Figure 5 presents the operational performance of the Tm:YAP PQS laser integrated with the ND-based SA. Depicted in Figure 5a is the output power versus absorbed pump power relationship for two OC transmission values (5% and 2%). With continuous-wave (CW) slope efficiencies of 21.4% (2% OC) and 19.6% (5% OC), the cavity yields respective PQS slope efficiencies of 3.2% and 3.6% from SA-induced loss. For both cases, the output power exhibits a linear rise with increasing absorbed pump power, confirming stable laser performance under the tested conditions. For CW operation at 9.66 W absorbed pump power, the output powers were measured to be 1.72 W (with 5% OC) and 1.68 W (with 2% OC). Following the insertion of the ND-based SA, the laser entered PQS mode, giving corresponding maximum average output powers of 367.7 mW and 334.8 mW. The output drops from 1.72 W (CW) to 367.7 mW (PQS). ND-SA introduces linear insertion loss and cyclic unsaturated nonlinear loss. Figure 5a shows the CW/PQS power gap barely narrows with increasing pump power, confirming linear scattering/absorption dominates efficiency degradation. A fast photodetector (ET-5000, EOT, Traverse City, MI, USA, rise time: 28 ps), together with a digital oscilloscope (MDO34, Tektronix, Beaverton, OR, USA, rise time: 3.5 ns), was employed to characterize the temporal pulse behavior, and the resulting dependence of pulse width and PRF on absorbed pump power is displayed in Figure 5b. When the absorbed pump power remained below 5.72 W, the pump energy was insufficient to drive significant population accumulation in the upper laser level of the Tm:YAP crystal, which in turn prevented the ND-based SA from being saturated by the intracavity laser field. As the pump power increased from 5.72 W to 9.66 W, the pulse parameters changed markedly. For the 2% OC, the pulse duration dropped from approximately 1250 ns to 440.1 ns, while the PRF rose from about 29.59 kHz to 84.21 kHz. For the 5% OC, the pulse width decreased from roughly 1122 ns to 376.9 ns, and the PRF increased from 45.15 kHz to 86.27 kHz. These observations indicate that higher pump power accelerates gain recovery and strengthens the saturation effect of the SA, thereby generating shorter pulses and higher repetition rates.

**Figure 6:**
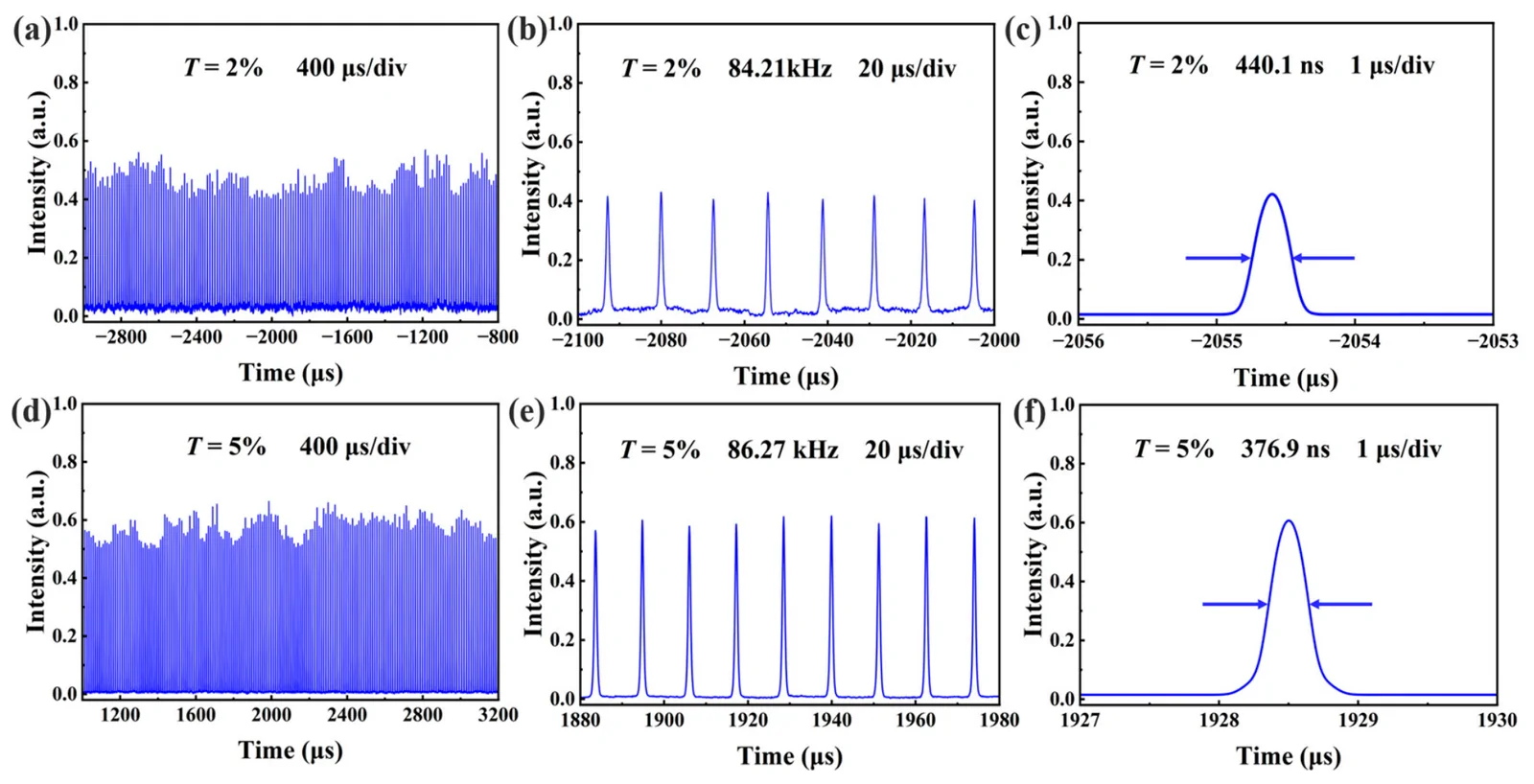
Stable pulse trains from the PQS Tm:YAP laser at *T* = 2% and 5%, shown at time scales of 400 μs/div, 20 μs/div, and 1 μs/div. (**a**) *T* = 2%, 400 μs/div; (**b**) *T* = 2%, 20 μs/div; (**c**) *T* = 2%, 1 μs/div; (**d**) *T* = 5%, 400 μs/div; (**e**) *T* = 5%, 20 μs/div; (**f**) *T* = 5%, 1 μs/div.

In the PQS Tm:YAP laser experiment, a stable pulse train was obtained with an OC transmittance of 2% (Figure 6a–c). At the 400 μs/div time scale, the laser operates stably. At a scale of 20 μs/div, a PRF of 84.21 kHz was clearly resolved, and the pulse intervals were uniform, confirming the high periodicity of the *Q*-switching process. Under a temporal resolution of 1 μs/div, the pulse duration was measured to be 440.1 ns. The symmetric, steep-edged waveform suggested that the ND film SA possessed excellent recovery characteristics, enabling effective pulse compression. As illustrated in Figure 5b, the pulse width decreased from 1260 ns to 376.9 ns as the absorbed pump power increased, demonstrating pulse compression in the PQS build-up. When the OC transmittance was increased to 5%, the laser maintained a stable pulsed output (Figure 6d–f). At both 400 μs/div and 20 μs/div scales, the pulse sequence remained steady, and the PRF rose to 86.27 kHz. At 1 μs/div resolution, the single-pulse duration was 376.9 ns, further verifying the pulse-shortening effect of the output coupling ratio. Overall, the ND film as an SA demonstrates outstanding PQS performance in the Tm:YAP laser. Stable nanosecond pulses can be generated under various output coupling conditions, with pulse duration decreasing and PRF increasing as the OC transmittance is raised. These results provide a reliable experimental foundation for the design of pulsed lasers.

**Figure 7:**
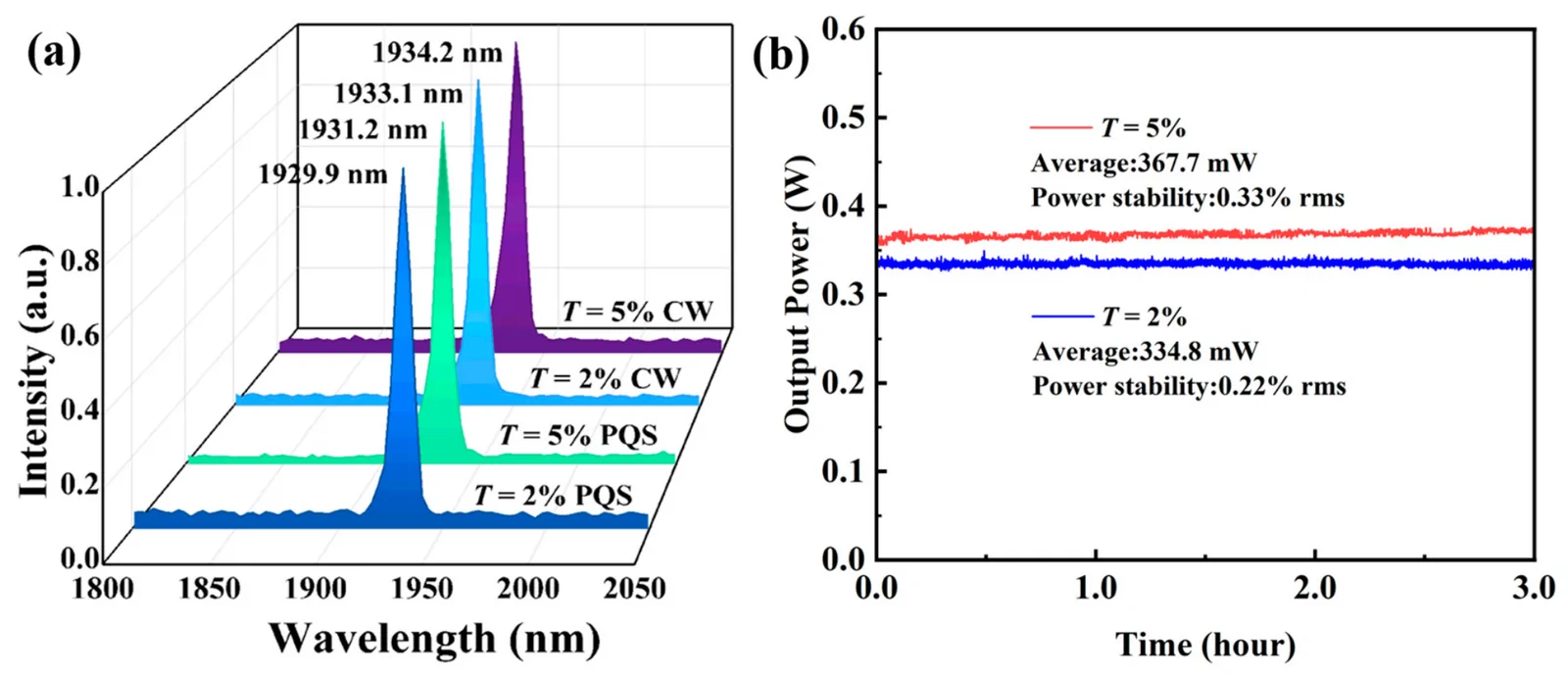
(**a**) Optical spectra of the Tm:YAP laser in CW and PQS modes. (**b**) Output power stability of the PQS Tm:YAP laser at *T* = 2% and 5%.

Figure 7a shows the laser spectra measured by an optical spectrum analyzer (SpectraPro HRS-500, Princeton Instruments, Trenton, NJ, USA) at different OC transmittances. All spectra exhibit a sharp single peak, confirming stable single-peak operation. Under CW mode, the central output wavelength is 1934.2 nm at *T* = 5% and 1933.1 nm at *T* = 2%. With the ND-based SA introduced for PQS, a clear blue shift of the central output wavelength occurs: it moves to 1931.2 nm at *T* = 5% and to 1929.9 nm at *T* = 2%. This blue shift is primarily ascribed to enhanced energy storage in the Tm:YAP crystal under PQS conditions, which drives the gain peak toward shorter wavelengths [7]. Figure 7b shows the stability of the PQS laser output power during 3 h of continuous operation, expressed as the relative root-mean-square (RMS) power fluctuation, i.e., the RMS deviation (calculated from power readings taken every second) divided by the mean output power. With an OC transmittance of 5%, the relative RMS fluctuation is 0.33% at an average output power of 367.7 mW. For *T* = 2%, this value drops to 0.22% with an average power of 334.8 mW. These findings demonstrate excellent power stability for the ND-SA based Tm:YAP pulsed laser.

**Figure 8:**
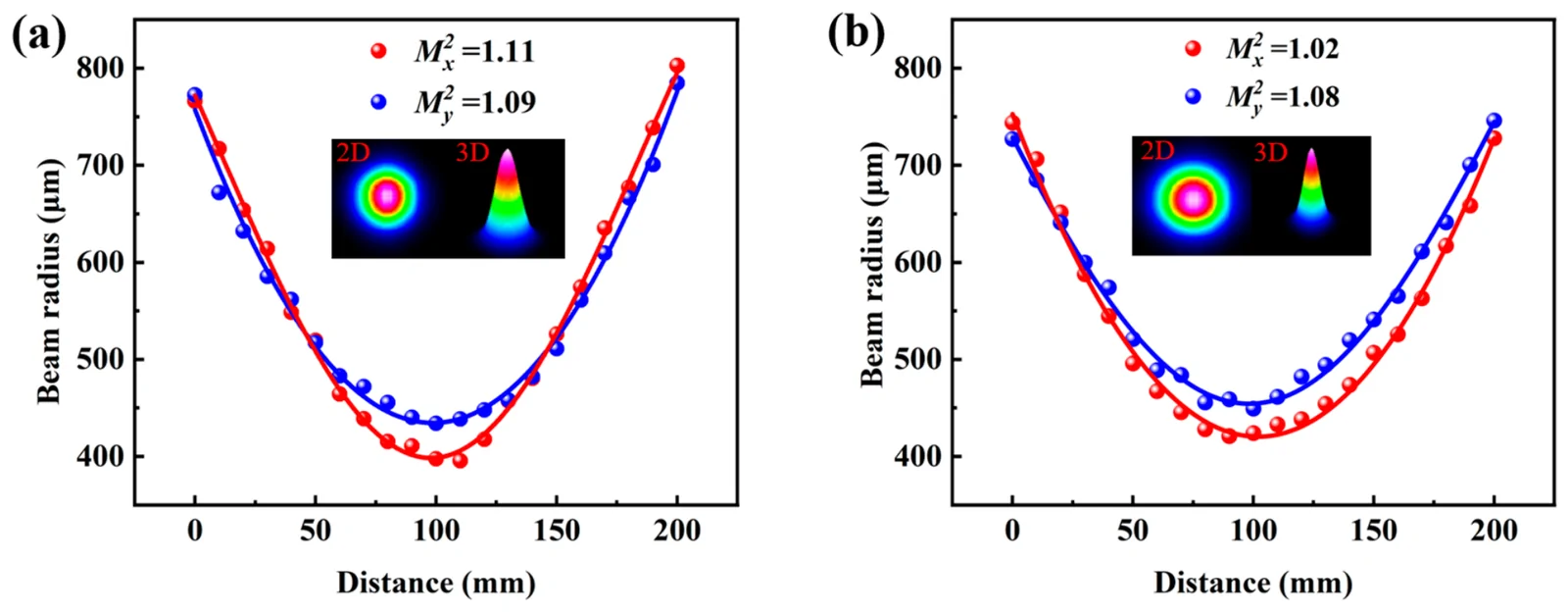
Beam quality of the PQS Tm:YAP laser measured at OC transmittances of (**a**) *T* = 2% and (**b**) *T* = 5%.

The beam propagation properties were measured using a beam quality analyzer (M2MS-BP209IP2, Thorlabs, Newton, NJ, USA) for OC transmittances of 2% and 5%. By fitting the measured beam radii to the standard Gaussian propagation equation (Figure 8a), the *M*^2^ factors were determined, revealing the spatial mode characteristics of the PQS Tm:YAP laser. At *T* = 5%, the beam quality factors in the horizontal and vertical directions reached $M_{x}^{2}$ = 1.02 and $M_{y}^{2}$ = 1.08, both close to the diffraction limit (*M*^2^ = 1) and substantially better than those at *T* = 2%. This indicates stable TEM_00_ operation at this output coupling level. The corresponding intensity distribution (inset of Figure 8b) shows a perfectly circular, symmetric Gaussian profile without any higher-order mode components or distortions. Such near-diffraction-limited beam quality provides excellent spatial coherence and propagation stability for the 2 μm laser, making it highly attractive for demanding applications.

Table 1 compares the output performance of PQS Tm:YAP solid-state lasers operating in the 2 μm eye-safe band with various SAs. All listed systems employ Tm:YAP as the gain medium, and the output wavelengths span from 1931.2 to 1995.4 nm. Among them, the laser based on the proposed ND-based SA stands out in terms of pulse width, achieving the minimum value of 376.9 ns among all compared results, significantly surpassing those using PZT, Nb_2_AlC, TaTe_2_, SnS_2_, NiFe-LDO, and V_2_GeC, thus showing potential for applications that demand high temporal resolution. Moreover, this laser delivers the shortest operating wavelength of 1931.2 nm in the comparison, effectively broadening the wavelength coverage of YAP PQS lasers while maintaining a competitive peak power of 11.33 W. As a newly developed high-performance SA material, NDs demonstrate excellent *Q*-switching capability, confirming their great promise for constructing narrow-pulse-width 2 μm pulsed solid-state lasers.

## 5. Conclusions

In conclusion, an ND-based SA enabled a diode-pumped PQS Tm:YAP laser to emit pulsed radiation at 2 μm. The spin-coated ND film demonstrated good uniformity and strong adhesion to the substrate. At 9.66 W absorbed pump power, the laser delivered an average output of 367.7 mW. Using a 5% transmittance OC, stable pulse trains were generated with a minimum pulse width of 376.9 ns at 86.27 kHz. The output wavelength centered at 1931.2 nm, exhibiting a distinct blue shift from the CW case. The beam quality approached the diffraction limit, with *M*^2^ factors of 1.02 (horizontal) and 1.08 (vertical). The achieved pulse width represents the shortest value reported to date for this laser configuration based on diamond-family SAs. Long-term power stability measurements indicated that the relative root-mean-square power fluctuation ranged from 0.22% to 0.33%, reflecting excellent operational stability. These results collectively demonstrate that NDs combine effective saturable absorption with high thermal stability and mechanical durability, making them a highly efficient near-infrared SA material particularly well-suited for 2 μm pulsed laser applications requiring both narrow pulse widths and high stability.

## Figures and Tables

**Table 1:** Comparative output performance of PQS solid-state lasers employing different SAs.

SA	Gain Medium	Output Wavelength (nm)	Average Output Power (W)	Pulse Width (ns)	Pulse Repetition Frequency (kHz)	Peak Power (W)	Single Pulse Energy (μJ)	Year	Reference
PZT	Tm:YAP	1991.9	0.81	1690	175	2.74	4.63	2021	[41]
Nb_2_AlC	Tm:YAP	1987.2	1.62	850	135	14.1	12	2022	[42]
TaTe_2_	Tm:YAP	1987.9	1.86	585	191.16	16.5	9.7	2023	[43]
SnS_2_	Tm:YAP	1988.29	0.621	3061	70.42	2.88	8.82	2023	[44]
NiFe-LDO	Tm:YAP	1931.2	0.425	519.1	106.9	7.65	3.97	2025	[45]
V_2_GeC	Tm:YAP	1937.4	0.663	416.7	129	12.3	5.1	2025	[46]
ND	Tm:YAP	1931.2	0.368	376.9	86.27	11.33	4.27	2025	This work

## Data Availability

The original contributions presented in this study are included in the article. Further inquiries can be directed to the corresponding author.

## References

[1] Li K., Niu C., Wu C., Yu Y., Ma Y. (2023). Development of a 2 μm Solid-State Laser for Lidar in the Past Decade. Sensors.

[2] Yang Y., Gao L., Han Y., Gao Q., Lan R., Shen Y. (2024). Passively Q-switched Tm:YAP laser based on WSe_2_/CuO heterojunction saturable absorber. Appl. Phys. B.

[3] Kang P., Zhang S., Zhang X., Huang J. (2022). High power self-Q-switched Tm:YAP solid state laser based on the ground state reabsorption. Infrared Phys. Technol..

[4] Wang Y., Ding K., Sun B., Lee S.T., Jie J. (2016). Two-dimensional layered material/silicon heterojunctions for energy and optoelectronic applications. Nano Res..

[5] Zhang X., Zhang Y., Xu R., Pan J., Hu L., Duan W., Zhao W. (2022). 2D self-assembled perovskite nanosheets as a nonlinear saturable absorber for passive Q-switched laser. J. Lumin..

[6] Shen J.P., Chen Y., Chen L., Xing F.Y., Zhang F.B., Xia R.Z., Zuo H.Y., Xiong F., Jiang R.R. (2025). A High Peak Power Passively Q-Switched Nd:YAG Dual-Rod 532 nm Laser Based on LED Side Pumping. Chin. Phys. Lett..

[7] Wang X., Long Z., Zhong W., Yang Y., Du A., Yang W., Sun C., Shen Y., Lan R. (2025). Passively Q-switched Tm:YAP laser based on MoS_2_/CuO hereojunction saturable absorber. Opt. Commun..

[8] Wang C., Peng Q.Q., Fan X.W., Liang W.Y., Zhang F., Liu J., Zhang H. (2018). MXene Ti_3_C_2_T_x_ saturable absorber for pulsed laser at 1.3 μm. Chin. Phys. B.

[9] Wang X., Xu J., Suh Y., Feng W., You Z., Sun D., Tu C. (2017). 2 and 3 μm passively Q-switched bulk pulse laser based on a MoS_2_/graphene heterojunction. Laser Phys. Lett..

[10] Cheng L., Wang X., Gong F., Liu T., Liu Z. (2020). 2D nanomaterials for cancer theranostic applications. Adv. Mater..

[11] Sun Y., Xia H. (2022). Bi_2_Te_3_/Graphene Heterostructure as the Saturable Absorber for ~1.0 μm Passively Q-switched Solid State Pulsed Laser. Crystals.

[12] Zhai X., Ding Y., Min H., Gao L., Liu G., Lan R., Shen Y. (2023). An infrared passively Q-switched laser based on graphene/BN heterojunction. Infrared Phys. Technol..

[13] Wang X., Xu J., Gao S., Sun Y., Zhu Z., You Z., Tu C. (2017). Dual-wavelength passively Q-switched bulk laser using MoS_2_/graphene heterojunction. Mater. Res. Bull..

[14] Lyon D., Hubler A. (2013). Gap size dependence of the dielectric strength in nano vacuum gaps. IEEE Trans. Dielectr. Electr. Insul..

[15] Yao B.Q., Cui Z., Duan X.M., Shen Y.J., Wang J., Du Y.Q. (2014). A Graphene-Based Passively Q-Switched Ho:YAG Laser. Chin. Phys. Lett..

[16] Xie G.Q., Ma J., Lv P., Gao W.L., Yuan P., Qian L.J., Yu H.J., Zhang H.J., Wang J.Y., Tang D.Y. (2012). Graphene saturable absorber for Q-switching and mode locking at 2 μm wavelength [Invited]. Opt. Mater. Express.

[17] Jariwala D., Sangwan V.K., Lauhon L.J., Marks T.J., Hersam M.C. (2013). Carbon nanomaterials for electronics, optoelectronics, photovoltaics, and sensing. Chem. Soc. Rev..

[18] Cui K., Maruyama S. (2019). Multifunctional graphene and carbon nanotube films for planar heterojunction solar cells. Prog. Energy Combust. Sci..

[19] Mohammed D.Z., Al-Janabi A.H. (2016). Passively Q-switched erbium doped fiber laser based on double walled carbon nanotubes-polyvinyl alcohol saturable absorber. Laser Phys..

[20] Tegafaw T., Liu S., Ahmad M.Y., Al Saidi A.K.A.A., Zhao D., Liu Y., Yue H., Nam S.W., Chang Y., Lee G.H. (2023). Production, surface modification, physicochemical properties, biocompatibility, and bioimaging applications of nanodiamonds. RSC Adv..

[21] Mochalin V.N., Shenderova O., Ho D., Gogotsi Y. (2012). The properties and applications of nanodiamonds. Nat. Nanotechnol..

[22] Shenderova O.A., McGuire G.E. (2015). Science and engineering of nanodiamond particle surfaces for biological applications (Review). Biointerphases.

[23] Vervald A.M., Burikov S.A., Shenderova O.A., Nunn N., Podkopaev D.O., Vlasov I.I., Dolenko T.A. (2016). Relationship Between Fluorescent and Vibronic Properties of Detonation Nanodiamonds and Strength of Hydrogen Bonds in Suspensions. J. Phys. Chem. C.

[24] Imam A., Boileau A., Gries T., Ghanbaja J., Mangin D., Hussein K., Sezen H., Amati M., Belmonte T. (2016). Nanostructures design by plasma afterglow-assisted oxidation of iron–copper thin films. J. Cryst. Growth.

[25] Angus J.C. (1991). Diamond and diamond-like phases. Diam. Relat. Mater..

[26] Power A.C., Gorey B., Chandra S., Chapman J. (2018). Carbon nanomaterials and their application to electrochemical sensors; a review. Nanotechnol. Rev..

[27] Vanyukov V., Mikheev G., Mogileva T., Puzyr A., Bondar V., Lyashenko D., Chuvilin A. (2017). Saturable absorption in detonation nanodiamond dispersions. J. Nanophoton..

[28] Mikheev G.M., Krivenkov R.Y., Mogileva T.N., Mikheev K.G., Nunn N., Shenderova O.A. (2017). Saturable Absorption in Suspensions of Single-Digit Detonation Nanodiamonds. J. Phys. Chem. C.

[29] Mikheev G.M., Vanyukov V.V., Mogileva T.N., Mikheev K.G., Aleksandrovich A.N., Nunn N.A., Shenderova O.A. (2021). Femtosecond Optical Nonlinearity of Nanodiamond Suspensions. Appl. Sci..

[30] Shakaty A.A., Hmood J.K., Mahdi B.R., Mahdi R.I., Al-Azzawi A.A. (2021). Q-switched erbium-doped fiber laser based on nanodiamond saturable absorber. Opt. Laser Technol..

[31] Wu Q., Wang Y., Zhan G., Wu H., Hu Y., Wang M. (2023). Graphdiyne-Based All-Solid-State Passively Q-Switched Tm:YAP Laser at 2 μm. Nanomaterials.

[32] Jin C.J., Bai Y., Li L.F., Jiang T., Ren Z.Y., Bai J.T. (2014). A single-frequency, graphene-based passively Q-switched Tm:YAP laser. Laser Phys..

[33] Wang Y., Ma Z., Bao Z., Zhao S.S., Hu B., Zhang H., Zhao J., Li X., Zhao Z. (2025). Environment-Friendly N-Doped Carbon Dots for Fingerprint and High Color-Rendering White Light-Emitting Diode. ChemistrySelect.

[34] Shakaty A.A., Hmood J.K., Mahdi B.R., Harun S.W. (2022). Passively mode-locked erbium-doped fiber laser based on a nanodiamond saturable absorber. Appl. Opt..

[35] Schrand A.M., Hens S.A.C., Shenderova O.A. (2009). Nanodiamond Particles: Properties and Perspectives for Bioapplications. Crit. Rev. Solid State Mater. Sci..

[36] Dolmatov V.Y., Lapchuk N.M., Lapchuk T.M., Nguyen B.T.T., Myllymäki V., Vehanen A., Yakovlev R.Y. (2016). A study of defects and impurities in doped detonation nanodiamonds by EPR, Raman scattering, and XRD methods. J. Superhard Mater..

[37] Ferrari A.C., Robertson J. (2004). Raman spectroscopy of amorphous, nanostructured, diamond-like carbon, and nanodiamond. Philos. Trans. R. Soc. A.

[38] Jorio A., Ferreira E.H.M., Moutinho M.V.O., Stavale F., Achete C.A., Capaz R.B. (2010). Measuring disorder in graphene with the G and D bands. Phys. Status Solidi B.

[39] Hu L., Guo Y., Du S., Tian S., Li J., Gu C. (2021). Probing trans-polyacetylene segments in a diamond film by tip-enhanced Raman spectroscopy. Diam. Relat. Mater..

[40] Papagiannouli I., Bourlinos A.B., Bakandritsos A., Couris S. (2014). Nonlinear optical properties of colloidal carbon nanoparticles: Nanodiamonds and carbon dots. RSC Adv..

[41] Li S., Li L., Qi T., Wu Z., Duan X., Yang X., Shen Y. (2021). Passively Q-switched Tm:YAP laser with a lead zirconate titanate saturable absorber. Appl. Opt..

[42] Hu Y., Yang X., Li L., Gao Q., Li S., Wu Z., Yang Y., Cui H., Zhou S. (2022). A passively Q-switched operation of Tm:YAP laser with a Nb_2_AlC-based saturable absorber. Optik.

[43] He X., Qi T., Gao Q., Yang W., Yu S. (2022). Passive Q-switched operation of Tm:YAP laser with a TaTe_2_ saturable absorber. Microw. Opt. Technol. Lett..

[44] Shi Z., Sun X., Xun J., Xie W., Zhou L. (2023). A diode-pumped SnS_2_ passively Q-switched tm-doped pulsed laser. Microw. Opt. Technol. Lett..

[45] Chen T., Wang C., Meng Z., Niu S., Li Z., Yang X. (2025). NiFe-layered double oxide as a saturable absorber for near-infrared passively Q-switched all-solid-state laser. Opt. Mater..

[46] Li H., Gao Q., Ma X., Qu X., Li L. (2025). Passively Q-switched Tm:YAP laser with a V_2_GeC saturable absorber. Appl. Phys. B.

